# Effect of Fatty Acids on Backfat Quality in Beijing Black Pigs

**DOI:** 10.3390/foods13233927

**Published:** 2024-12-05

**Authors:** Xueli Zhu, Weilong Tian, Ziping Hu, Renda Hou, Xinhua Hou, Ligang Wang, Longchao Zhang, Lei Pu, Lixian Wang, Xin Liu

**Affiliations:** 1 Institute of Animal Science, Chinese Academy of Agricultural Sciences, Beijing 100193, China; zhuxueli199907@163.com (X.Z.); hu07232021@163.com (Z.H.); 15650199601@163.com (R.H.); 7hxh73@163.com (X.H.); ligwang@126.com (L.W.); zhlchias@163.com (L.Z.); iaswlx@263.net (L.W.); 2 Tianjin Key Laboratory of Agricultural Animal Breeding and Healthy Husbandry, College of Animal Science and Veterinary Medicine, Tianjin Agricultural University, Tianjin 300392, China; pulei87@126.com; 3 Chizhou Vocational and Technical College, Guichi District, Chizhou 247100, China; twl2103755661@163.com

**Keywords:** backfat quality, fatty acid, candidate genes, Beijing Black pig

## Abstract

The quality of pig backfat affects both pork quality and consumer preferences. Fatty acids (FAs) are crucial in determining the backfat quality. This study assessed the effect of FAs on the backfat quality and identified candidate genes associated with these FAs. The differential fatty acids (DFAs) were compared in pigs with varying backfat firmness and four DFAs—caproic acid, stearic acid, linoleic acid and alpha-linolenic acid—were selected based on T-tests (*p* < 0.05), fold changes (FC > 2 or FC < 0.5), and variable importance (VIP > 1). Genome-wide association studies on the DFAs and linoleic acid/alpha-linolenic acid ratios in 413 Beijing Black pigs identified 22 single-nucleotide polymorphisms significantly associated with one or more traits. The genes *PLPP3*, *MGLL*, *CYP27A1* and *UBE3C* were identified as candidates associated with these traits influencing the backfat quality. These findings enhance our understanding of the backfat quality in Beijing Black pigs and provide a basis for further research.

## 1. Introduction

China is a major global producer and consumer of pork, with pork production accounting for over 50% of the total meat production [1]. As living standards improve, consumers are more inclined to look for tastier, safer and more nutritious pork. Consequently, there is growing emphasis on the quality of pork [2]. The most distinctive feature of fresh pork is its appearance, which is influenced by many factors, including the animal’s diet, age and genetics [3]. Fat quality plays a crucial role in determining the overall quality of pork and its products, influencing both their nutritional value and organoleptic properties [4]. Despite its importance, fat quality has received less attention compared to other meat quality attributes such as pH and drip loss [5]. From a consumer perspective, white fat in the backfat of a carcass is generally preferred over yellow fat. Researchers have classified white and firm fats as indicators of high quality, while fats that are soft, greasy, gray or yellow are considered of lower quality [6]. Furthermore, fat firmness is crucial for the food processing industry [7]. Softer fats are more easily dissolved and lost during processing, which could adversely affect the product’s drying performance [8].

Fatty acids (FAs) are the primary component of fat, and their composition and physical properties are important factors influencing the firmness of pig backfat [9,10]. FAs are a type of lipids, and in the diet, FAs are divided into three main categories: saturated fatty acids (SFAs), monounsaturated fatty acids (MUFAs) and polyunsaturated fatty acids (PUFAs) [11]. The composition and content of animal FAs vary among species and fat tissues [12]. Local pig breeds tend to have a higher proportion of MUFAs and SFAs [13]. Additionally, it has been shown that the FA composition and content is also influenced by genetic factors and further affects meat quality. The heritability estimates for certain FAs, including palmitic acid (PA, C16:0), stearic acid (SA, C18:0), and oleic acid (OA, C18:1), have been reported in the range of 0.15–0.47, suggesting that FA content traits are genetically influenced [14]. The FA metabolism pathway is different in terms of the transcriptome level in Bama pigs and Gansu black pigs, which led to a difference in meat quality [15]. In Ningxiang pigs, transcriptomic and metabolomic analyses revealed eight differentially expressed genes and three significantly altered metabolites, including arachidonic acid (ARA), OA and linoleic acid (LA), at different developmental stages [16]. A functional enrichment analysis of differential genes obtained from the transcriptome sequencing of the back adipose tissue in Duroc and Luchuan pigs showed that variations in the fat deposition may be attributed to differences in the production pathways of α-linolenic acid (ALA), LA, and ARA, which could further influence the flavor profiles of these pork types [17].

The fat quality of pig backfat has a significant impact on pork and product quality, yet it receives insufficient attention and there are few studies on it currently. And although some studies have focused on the impact of differential fatty acid (DFA) compositions and contents on meat quality, the specific effects of FAs on backfat quality and the underlying genetic mechanism remain unclear. Therefore, research on pig backfat quality, particularly FAs’ effect on it is urgently needed. The Beijing Black pig, a distinguished local breed primarily raised in Northeast and North China, is renowned for its firm, tender and adaptable meat characteristics [18]. Based on the existing Beijing Black pig population, this study was conducted to investigate the effect of the differential FA composition and content of the backfat between extremely different groups of backfat quality and further research on the genetic mechanisms of these influential FAs. The findings of this research will provide valuable insights and references for improving both the meat and backfat quality in Beijing Black pigs.

## 2. Materials and Methods

### 2.1. Animals and Sample Collection

In this study, 413 Beijing Black pigs were used from a pig industry company in Beijing. All the pigs were healthy and raised in unified feeding and management conditions. All test pigs were fed with a fattening diet produced by Da Bei Nong Group. The ratio of sows and castrated boars was approximately 1:1, and the slaughtering age was about 210 days old, with an average slaughter weight of about 90 kg. All were fasted for one day before slaughter, with free access to water, and then slaughtered after electrical stunning. The back adipose tissue was collected from the left ketone body after slaughter. The fat samples were partially placed in a ziplock bag = stored in the refrigerator at 4 °C for the measurement of fat firmness and 24 h fat color phenotype. The remaining backfat tissue was flash frozen in liquid nitrogen and stored at −80 °C for subsequent determination of fatty acid content and genome analysis. All experimental protocols were conducted in accordance with the guidelines, which were approved by the Ethics Committee of the Institute of Animal Sciences of the Chinese Academy of Agricultural Sciences.

### 2.2. Backfat Quality and FA Content Measurement

Backfat color and firmness were measured at 24 h post-mortem using established methods [19]. The color of backfat was measured using a Minolta CR200 colorimeter (Minolta Camera, Osaka, Japan) on the surface of the fat. The measurement followed the manufacturer’s recommended procedure. The CIE L* a* b* system was employed to evaluate the pork color, where L* indicated lightness, a* represented redness and b* represented yellowness. Among these parameters, the b* value, which reflected the yellowness of the fat surface, was selected as the primary phenotypic trait for assessment [20]. The firmness of adipose tissue was determined by TA-XT2 texture analyzer (Stable Micro Systems Ltd., Surrey, UK). The peak force (Newton, N) required to compress the fat was recorded as an indicator of fat firmness, serving as another critical phenotypic trait.

The content of 32 common FAs in backfat tissues was determined using gas chromatograph GC7890A (Agilent Technologies, Santa Clara, CA, USA) with a DB-23 capillary column (60 m × 0.25 mm i.d., 0.25 μm film thickness) coupled with a flame-ionization detector (Agilent Technologies, Santa Clara, CA, USA). Sample pre-treatment: Approximately 0.1 g of backfat tissue, with as much fascia as possible removed, was weighed into a screw-top glass vial and 1.5 mL of methyl undecanoate hexane internal standard was added. After mixing, 2.0 mL of 0.5 mol/L potassium hydroxide methanol was added, which was followed by vortex mixing and incubation in a 95 °C water bath for 10–30 min. After cooling to room temperature, 2.0 mL of 14% boron trifluoride methanol was added, followed by vortex mixing and incubation in a 95 °C water bath for an additional 10–30 min. After cooling to room temperature, 5 mL of saturated sodium chloride solution was added, and the mixture was vortexed and centrifuged at 2000 rpm for 3 min. More than 1 mL of the supernatant was taken and filtered through an organic membrane filter, and then put in the machine to be measured. GC parameters were as follows: injection volume, 10 µL; column flow rate, 1.2 mL/min; detector temperature, 250 °C. The temperature gradient was as follows: 50 °C for 2 min, then, at a rate of 25 °C/min, this increased to 175 °C for 3 min, followed by increasing at 5 °C/min to 200 °C, followed by increasing at 1.5 °C/min to 210 °C, then increasing at 2 °C/min to 230 °C for 6 min. The FAs were identified by comparison of peak retention times with those of authentic standards. FA identification was performed using standards, with methyl undecanoate as the internal standard.

Based on phenotypic data of the population, 15 samples exhibiting higher firmness and a lower 24 h b-value were selected as the high-quality group (HG), while 15 samples with lower firmness and a higher 24 h b-value were classified as the low-quality group (LG). The phenotypic values between the two groups were significantly different, with differences > 2 standard deviations. These samples were subsequently used to determine the FA content. A significant difference analysis was conducted to identify differential fatty acids (DFAs) between the two groups. Finally, the identified DFAs were detected in the population.

### 2.3. Genome-Wide Association Study (GWAS) on DFAs

DNA was extracted from all test Beijing Black pigs, and the GeneSeek Genomic Profiler (GGP) Porcine 50K chip (Illumina, San Diego, CA, USA) was applied to carry out single-nucleotide polymorphism (SNP) typing for test population. PLINK v1.90 was utilized for quality control (QC) according to the following criteria were retained: SNPs with a detection rate higher than 90%, a minor allele frequency (MAF) higher than 5%, individuals with a genotype detection rate higher than 90%, Hardy–Weinberg equilibrium (HWE), *p* < 0.5. Principal component analysis (PCA) was performed on the genome using NovoMagic (https://magic.novogene.com/customer/main#/loginNew, accessed on 1 March 2024), and the first three principal component effects (PCs) were used as covariates in GWAS. We used the GCTA software (Version 1.93.3beta) [21] to perform a GWAS analysis using the mixed model:

y = Wα + Xβ + μ + ε

where y is the phenotypic value vector; W is the fixed effects matrix; α is the corresponding coefficient including the intercept; X is the genotype matrix marked on the tested gene locus; β is the labeled effect size; μ is a random multi-effect; ε is the residual error vector.

We used the false discovery rate (FDR), which means the expected proportion of true null hypotheses within the class of rejected null hypotheses, [22,23] to confirm the suggested threshold values, and the FDR was set to 0.01. The threshold P(FDR) was computed as follows:

P(FDR) = FDR × n/m

in which n represents the number of SNPs with *p* < 0.01, whereas m denotes the total number of SNPs. We also generated Manhattan plots and quantile–quantile (Q-Q) plots using the R software package (Version 4.0.2) [24].

### 2.4. Candidate Gene Screening and Marquee Gene Enrichment Analysis

Based on the GWAS results, SNPs significantly associated with DFAs were identified. Candidate genes located within 1 Mb upstream and downstream of these significant SNPs were retrieved from Ensembl (https://www.ensembl.org/index.html?redirect=no).

To further investigate these genes, enrichment analysis was performed using the Kyoto Encyclopedia of Genes and Genomes (KEGG) pathway-related database through OmicShare tools, a free online platform for data analysis (www.omicshare.com/tools), with a significance threshold set at *p* < 0.05. Additionally, Gene Ontology (GO) enrichment of the candidate genes was conducted also using OmicShare tools. For functional queries and literature review of candidate genes, the Genecards database (https://www.genecards.org/Search) was consulted.

### 2.5. Data Analysis

FA phenotype data were calculated using Excel 2016 and FA content (X) in the sample was expressed as mass fraction in grams per hundred grams (g/100 g). The computational formula was as follows:

Xi = (Ci × f × FFAME−FA))/(m⁄1.5 × d) × 0.0001

where Xi is the content of each FA in the sample in grams per 100 g (g/100 g); Ci is the concentration of each fatty acid methyl ester in the sample solution calculated by the standard curve, with units of µg/mL; f is the proportion of each FA in the total standard solution; FFAME-FA is the conversion coefficient of each fatty acid methyl ester into fatty acid; m is sample quality, with units of (g); 1.5 is the amount of the inner target added, with units of (mL); 0.0001 is the conversion factor to convert the value to units of per 100 g of sample.

The data of the two groups were subjected to one-way statistical analysis using T-test (Student’s *t*-test) to calculate *p*-values (*p* value) of various FA contents. The fold change (FC) method was used to calculate the difference multiple of the FA content. The variable importance in projection (VIP) value of the FA content was calculated by using the Orthogonal Partial Least Squares Discriminant Analysis (OPLS-DA) method. The data quality was analyzed using MetaboAnalyst (https://metaboanalyst.ca/). Based on the above statistical results, significant DFAs of backfat between HG and LG were determined with the following criteria: *p* < 0.05, FC > 2 or FC < 0.5, VIP > 1.

## 3. Results

### 3.1. Phenotypic Data Collation

**Figure 1:**
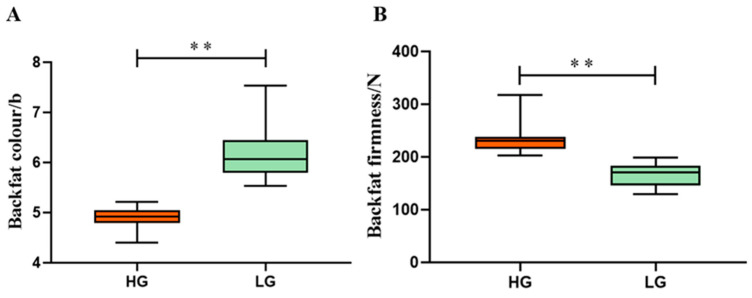
Comparison of differences in backfat quality between HG and LG. (**A**) The differences in the 24 h b-value between the two groups; (**B**) the differences in backfat firmness between the two groups. ** indicates significant differences at *p* < 0.01.

The phenotypic statistics for the backfat quality in the test population are provided in the Supplementary Materials (Table S1). A comparison between the HG and LG revealed notable differences. The mean value of firmness in the HG was 236.26 N, which was approximately 71.56 N higher than the mean value of firmness in the LG. Additionally, the mean value of the fat color (24 h b) in the HG was 4.88, which was about 1.3 units lower than in the LG. The phenotypic differences between the two groups were statistically significant (Figure 1).

A statistical analysis of 32 FA phenotypes of backfat was conducted between the HG and LG. The FA content was measured as the grams of fatty acid per 100 g of back adipose tissue (g/100 g). The results showed that the mean FAs in the HG ranges from 0.01 to 14.64 g/100 g, whereas, in the LG, the mean FA content ranged from 0.02 to 8.24 g/100 g. The details of the phenotypic statistics are shown in the Supplementary Materials (Table S2).

### 3.2. Multivariate Analysis of DFAs Between HG and LG

**Figure 2:**
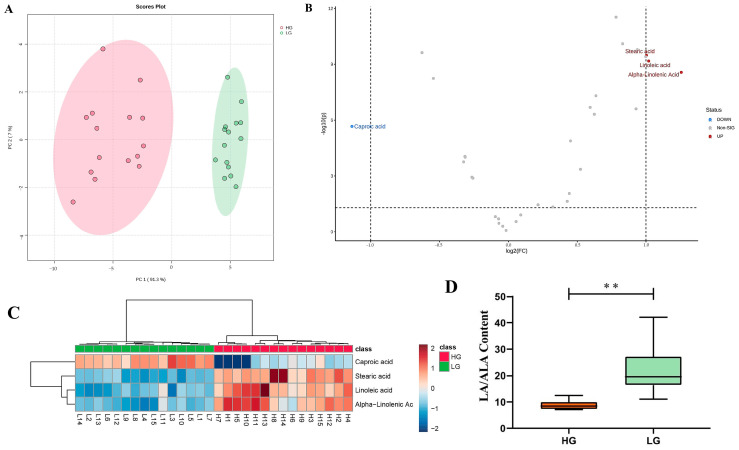
(**A**) PCA results for HG and LG; (**B**) volcano plot for DFAs; (**C**) hierarchical cluster analysis of the HG and LG; (**D**) the phenotypic values of LA/ALA for the HG and LG. ** indicates significant differences between the groups at *p* < 0.01.

The differences in various FA contents between the HG and LG were assessed. The PCA clearly separated the FAs of the HG and LG (Figure 2A), with PCA1 explaining 91.3% of the original data.

A differential analysis of 32 FAs between the HG and LG was performed using a significance threshold of *p* < 0.05. Out of these, 25 FAs met the criterion. Further screening using FC cutoffs (FC > 2 or FC < 0.5) identified LA, ALA, SA and caproic acid (CA) as being differentially expressed. Among these, LA, ALA and SA were significantly upregulated in the HG, while CA was significantly downregulated. To enhance the reliability, DFAs with VIP > 1 were selected using the OPLS-DA model. The final set of DFAs was determined by intersecting the results from three criteria (*p* < 0.05, VIP > 1, FC > 2 or FC < 0.5), yielding four DFAs: LA, ALA, CA and SA, in which SA and CA were SFAs, LA was a type of n-6 PUFA and ALA was a type of n-3 PUFA. The comparison results are presented in volcano maps and a hierarchical cluster analysis (Table 1 and Figure 2B,C).

Previous studies have emphasized the significant influence of the n-6/n-3 PUFA ratio on meat quality [25]. Given that LA and ALA were identified as significant DFAs between the HG and LG, we analyzed the ratio of LA/ALA to further evaluate the backfat quality. The results revealed that the mean LA/ALA ratio in the HG was 8.89, while, in the LG, it was 21.98. This difference indicated that the LA/ALA ratio in the HG was significantly lower than that of the LG (*p* < 0.01) (Figure 2D).

### 3.3. GWAS Analysis on DFAs

To further explore the genetic mechanisms underlying the DFAs, LA, SA, CA, ALA and LA/ALA phenotype detection and GWAS analyses were performed on a population of 413 Beijing Black pigs. The determination results of the DFA phenotype values in the test population showed that the means of the LA, SA, CA, ALA and LA/ALA values were 9.98, 8.02, 0.02, 0.5 and 20.90 g/100 g.

**Figure 3:**
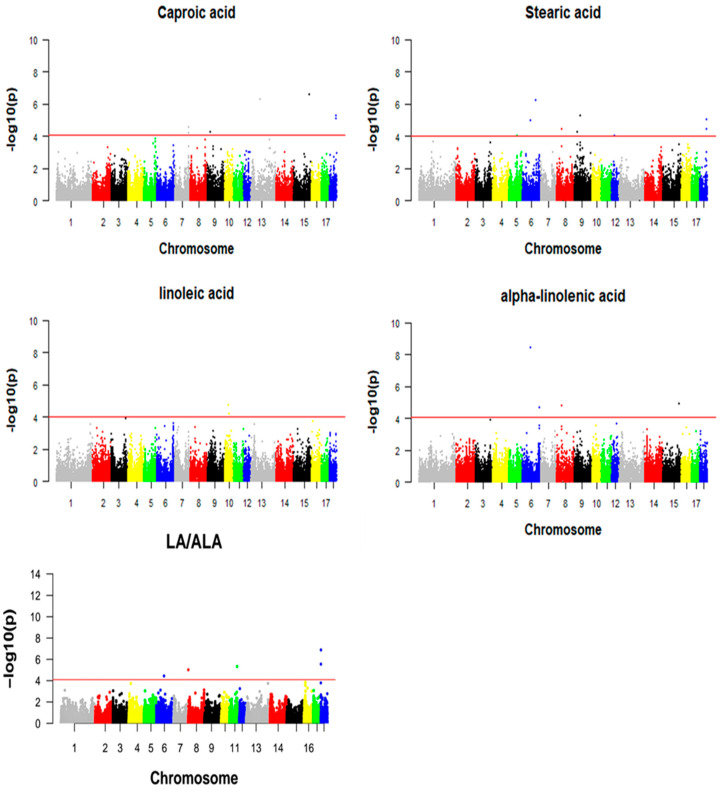
Manhattan map of DFAs of Beijing Black pigs.

The GWAS results for LA, SA, CA, ALA and LA/ALA showed a total of 21 suggestive significant SNPs associated with one or more DFAs, which are visualized by Manhattan plots (Figure 3). The significant SNPs associated with DFAs and their closet genes were annotated and are presented in Table 2.

Among them, seven SNPs on SSC 7, 9, 13, 15 and 18 were significantly associated with CA, and five genes were annotated. The SNP WU_10.2_15_134517097 on SSC15 showed the strongest association with CA and was annotated within the gene *OBSL1*. There were eight significant SNPs on SSC 5, 6, 8, 9, 12 and 18 associated with SA. The SNP ASGA0091446 on SSC 6 had the strongest association with SA and was located in the *IGSF21* gene. Two significant SNPs on SSC10 were found to be associated with LA. The SNP WU_10.2_10_33859597 was the most significant SNP associated with LA, and located near the *NTRK2* gene. Four significant SNPs on SSC6, 8 and 15 were found to be associated with ALA. The SNP ASGA0091446 on SSC 6, as the most significant SNP associated with ALA, was annotated on the *IGSF21* gene. Five significant SNPs on SSC6, 8, 11 and 18 were significantly associated with LA/ALA according to the GWAS. The most significant SNP, WU_10.2_18_1597750, of LA/ALA was annotated on the *UBE3C* gene.

### 3.4. Functional Analysis of Candidate Genes

To further investigate the candidate genes associated with the DFAs, we annotated genes within the 1 MB region upstream and downstream of the significantly associated SNPs using the Ensembl platform (https://www.ensembl.org/index.html?redirect=no). A total of 295 genes were annotated, including 122 genes associated with CA, 122 genes with SA, 10 genes with LA, 68 genes with ALA and 63 genes associated with the LA/ALA ratio. And also, some genes were annotated for multiple traits together (Table S3).

**Figure 4:**
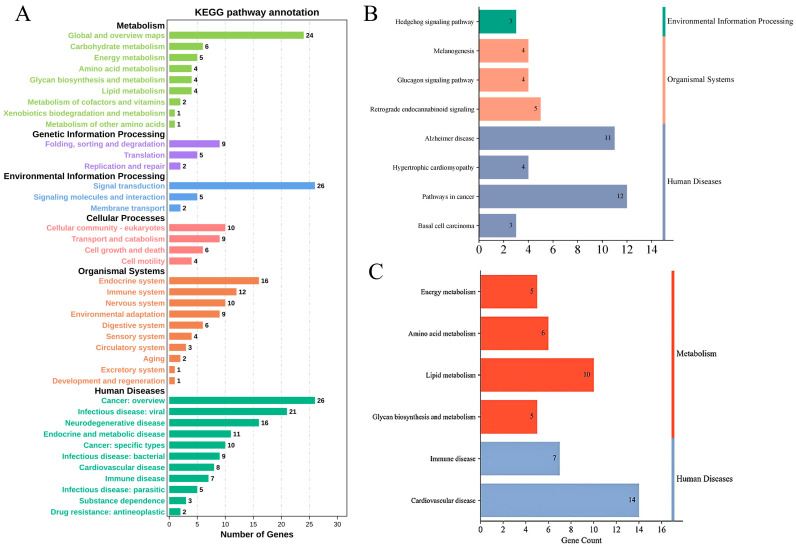
KEGG pathway analysis. (**A**) Enrichment pathways; (**B**) significant enrichment pathways (*p* < 0.05); (**C**) lipogenesis- and metabolism-related pathways.

Then, these annotated genes were subjected to a KEGG pathway enrichment analysis by using OmicShare tools, a free online platform for data analysis (www.omicshare.com/tools) (Figure 4A). The results identified 32 genes that were significantly enriched in nine pathways (*p* < 0.05) (Figure 4B, Table S4). The relevant functional pathways showed that 19 genes were significantly enriched in energy metabolism, amino acid metabolism, glycan biosynthesis metabolism and lipid metabolism pathways; 14 genes were significantly enriched in immune system pathways; and 8 genes were significantly enriched in cardiovascular disease pathways (Figure 4C). The functional annotation of genes was conducted for our concerned pathway (Table S5). Then, we found that phospholipid phosphatase 3 (*PLPP3*) regulated adipocyte sphingolipid synthesis; the enzyme monoglyceride lipase (*MGLL*) hydrolyzed triacylglycerol (TG) stored in adipocytes into fatty acids; and glycerol and the cytochrome P450 family 27 subfamily A member 1 (*CYP27A1*) gene encoded a cytochrome P450 enzyme. These genes were listed as candidate genes for influencing the backfat quality.

**Figure 5:**
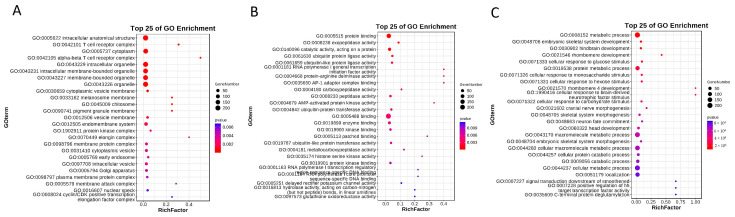
GO analysis bubble chart. (**A**) Cellular component, CC; (**B**) molecular function, MF; (**C**) biological process, BP.

Similarly, a GO analysis was performed on the preliminarily screened genes and only the top 25 biological processes with the strongest correlations were selected for visualization. The cellular component analysis showed that multiple genes were significantly associated with the cell receptor complex, T cell receptor complex, cytoplasmic structure, etc. (*p* < 0.05) (Figure 5A). The molecular function analysis identified significant associations with protein binding, exopeptidase activity, and catalytic activity on a protein, among others (*p* < 0.05) (Figure 5B). The biological process analysis showed that multiple genes were significantly correlated with metabolic processes, embryonic skeletal system development, hindbrain development, etc. (*p* < 0.05) (Figure 5C). However, no enrichment related to fat deposition and metabolism was identified in the GO analysis.

## 4. Discussion

FAs play crucial roles in the structure and quality of fat and meat [10]. Key meat characteristics influenced by FAs include fat firmness and flavor [26]. The effect of FAs on firmness is primarily attributed to their varying melting points. The different types and contents of FAs result in varying degrees of meat softness and firmness. The color change in meat occurs mainly due to rancidity reactions and lipid oxidation, which can promote pigment oxidation. UFAs are particularly susceptible to oxidative rancidity [27]. While the effects of FAs on meat quality are well documented, their impact on the backfat quality in pigs has not been extensively studied. In this study, we analyzed and compared the FA content in the backfat of Beijing Black pigs, which were categorized into HG and LG based on extreme differences in the backfat quality. Our results identified four DFAs (CA, SA, LA and ALA) that were significantly associated with the observed differences in the backfat quality.

The four DFAs identified in this study each have significant physiological functions and may substantially impact fat quality. CA, a type of short-chain fatty acid (SCFA), is commonly found in dairy products and in the human diet. CA is associated with the distinctive “goaty flavor” in goat milk, which can negatively affect consumer acceptance [28,29]. Additionally, CA has been shown to increase plasma and liver cholesterol concentrations, as well as blood glucose levels in mice [30]. Consequently, a lower proportion of CA in meat or backfat may be more conducive to meat flavor and health. SA, which is an SFA, is widely distributed in nature and is more readily absorbed by the body. SA metabolism is crucial for pork quality, as SFAs are chemically stable and less prone to oxidation [15]. Studies have demonstrated that a higher SA content is associated with the increased firmness of adipose tissue, enhancing meat firmness [26]. Our results showed that CA was present at a lower content, while SA was significantly upregulated in the HG, which reflected that the HG may exhibit a more favorable distribution of FAs, which contributes to the improved meat and fat quality.

LA and ALA, as typical n-6 and n-3 PUFAs, respectively, are essential FAs for mammals and must be obtained from the diet because they are not self-synthesized [31]. The excessive intake of LA and insufficient ALA can lead to an elevated n-6/n-3 ratio, which has been associated with the development of obesity [25]. LA can be converted into arachidonic acid (ARA), while ALA can be converted into eicosapentaenoic acid (EPA) and docosahexaenoic acid (DHA). EPA and DHA can reduce adipogenesis by inhibiting peroxisome proliferator-activated receptor γ (PPARγ) [32]. It has been shown that the addition of ALA inhibited cholesterol and FA synthesis in 3T3-L1 adipocytes through the suppression of the expression of Sterol regulatory element binding proteins (SREBPs) and fatty acid synthases [33]. Another aspect of research has shown that there is an effect on meat color b-values with dietary supplementation with flaxseed enriched with n-3 PUFAs [34]. In addition, a low n-6/n-3 PUFA ratio in pig diets can improve the meat color and enhance the meat quality [35]. Thus, the n-6/n-3 ratio not only influences adipogenesis but also acts on fat color. Our study’s results showed that there was a significant difference in the ratio of LA/ALA between the HG and LG, with a lower ratio observed in the HG. This suggests that the n-6/n-3 ratio may influence the backfat quality.

The FA composition and content determine the quality of fat, and also reflect the nutrition profile and quality of pork [36]. However, in most pig breeding farms, FA traits are not routinely measured, as these traits are not part of standard breeding metrics and are difficult to assess accurately. Consequently, traditional breeding strategies are not well suited for breeding FA traits. Previous studies have estimated the heritability of various FAs, revealing moderate to high heritability for most traits, suggesting that genetic components significantly contribute to FA traits [37]. Therefore, identifying genetic variations associated with FAs could aid in the development of molecular breeding strategies to enhance pork and fat quality. In this study, a GWAS was performed for the FA content of backfat and some candidate genes were explored. Through a further literature review of the genes located near the significantly associated SNPs, it was found that the *UBE3C* gene, which was the third enzyme in the protein ubiquitination pathway, directed the synthesis of ubiquitin protein ligase E3C (UBE3C) [38]. The E3 ubiquitin protein is involved in lipid deposition and metabolism, energy homeostasis, insulin resistance and other processes in mammals [39,40,41]. The *UBE3C* gene, located on SSC18 near the quantitative trait loci (QTLs) of intramuscular fat content (IMF) and FA composition, could be considered a candidate gene for pig fat deposition. Previous studies have reported SNP polymorphisms in *UBE3C* associated with the content of IMF and FAs, and some significantly associated candidate SNPs were identified [42,43]. In this study, the SNP located in *UBE3C* was significantly associated with the LA/ALA ratio. Combined with existing reports, our findings suggest that *UBE3C* and its associated SNPs may represent the important function genes or loci for FA traits.

Based on the KEGG pathway enrichment and gene function query, we focused on the role of the *PLPP3*, *MGLL*, and *CYP27A1* genes in adipogenic metabolism. *PLPP3*, which encodes lipid phosphatase (LPP) 3, was found to regulate the synthesis of adipocyte sphingolipids but did not ameliorate diet-induced obesity in mice with the inactivation of PLPP3 adipocyte targeting [44]. A high-fat diet and cardiac dysfunction were found to be associated with elevated LPP3 in mice fed a high-fat diet, and increased LPP3 contributes to insulin resistance by increasing the diacylglycerol (DAG) levels [45]. Li’s report showed that the addition of additives in the diets of yellow catfish inhibited the expression of PLPP3 while reducing the TG and total cholesterol (TC) contents, which affected the reduction in fatty deposition [46]. These studies suggest that *PLPP3* may play an important role in lipid synthesis, although the underlying mechanism remains complex.

Another candidate gene, *MGLL*, has been identified in expression-based genome-wide association studies (eGWASs) as a potential regulator of fat growth traits in pigs [47]. MGLL works with hormone-sensitive lipase to hydrolyze triacylglycerol (TG) stored in adipocytes into fatty acids and glycerol [48]. Its role in lipid metabolism makes it a significant candidate gene for studying fat deposition and meat quality traits in livestock.

CYP27A1 is a type of cytochrome P450 enzyme required for the biosynthesis of bile acids from cholesterol and is involved in the degradation of cholesterol in the liver [49]. Studies have shown that CYP27A1 metabolizes cholesterol into 27-hydroxycholesterol (27HC), the main cholesterol metabolite with anti-adipogenic activity in adipocytes, and that the presence of 27HC prevents adipose tissue expansion [50]. Studies in mice have also shown that the high intake of dietary n-3 PUFAs resulted in increased levels of CYP27A1 expression in the liver and brain [51].

In summary, previous studies have shown that these four genes influence fatty acid metabolism, lipogenesis and metabolic processes. However, their specific mechanisms related to the composition and content of FAs and even fat quality are not clear. Further research is needed to elucidate these mechanisms.

## 5. Conclusions

In this study, four DFAs (CA, SA, LA and ALA) were identified through a comprehensive analysis of the FA content between the HG and LG. The four DFAs and the LA/ALA ratio were analyzed by a GWAS in a population and 22 significantly associated SNPs were identified. Following pathway enrichment and gene annotation, the *UBE3C*, *PLPP3*, *MGLL* and *CYP27A1* genes were identified as candidate genes related to DFAs and may further influence the backfat quality. These findings lay the foundation for the further elucidation of the genetic mechanism of FAs and backfat quality variation in the Beijing Black pig.

## Figures and Tables

**Table 1:** DFA phenotypic value.

	DFAs	HG Mean ± sd	LG Mean ± sd	*p*-Value
1	Linolenic acid	9.43 ± 1.58	4.65 ± 1.10	2.02 × 10^10^
2	Alpha-linolenic acid	0.55 ± 0.11	0.23 ± 0.06	2.36 × 10^10^
3	Stearic acid	7.73 ± 1.16	3.85 ± 0.50	1.97 × 10^12^
4	Caproic acid	0.02 ± 0.01	0.03 ± 0.01	2.11 × 10^7^

The unit of fatty acid content is composed of fatty acid content (g) per 100 g of back adipose tissue, g/100 g.

**Table 2:** Significantly associated SNPs and annotated genes.

DFAs	Chr	SNP Name	SNP Position	*p*-Value	Annotated Gene *	Relationship Between SNP and Annotation Gene Location
CA	15	WU_10.2_15_134517097	121575947	2.80 × 10^7^	Obscurin like cytoskeletal adaptor 1 (*OBSL1*)	Intragenic
	13	ASGA0093606	73216491	5.44 × 10^7^	Coiled-coil-helix-coiled-coil-helix domain containing 6 (*CHCHD*6)	Intragenic
	18	WU_10.2_18_60133742	54860187	5.69 × 10^6^	POU class 6 homeobox 2 (*POU*6*F*2)	Intragenic
	18	WU_10.2_18_60174495	54904058	8.71 × 10^6^	*POU*6*F*2	Intragenic
	7	WU_10.2_7_119230503	112657325	2.82 × 10^5^	ribosomal protein S6 kinase A5(*RPS6KA5*)	Intragenic
	9	WU_10.2_9_25562285	23025287	5.85 × 10^5^	folate hydrolase 1B (*FOLH1B*)	Upstream of gene
	7	WU_10.2_7_119093281	112611525	6.39 × 10^5^	*RPS*6*KA*5	Intragenic
SA	6	ASGA0091446	76942191	3.91 × 10^9^	Immunoglobin superfamily member 21 (*IGSF*21)	Intragenic
	18	WU_10.2_18_60133742	54860187	5.69 × 10^6^	*POU*6*F*2	Intragenic
	9	ASGA0101263	45448558	5.92 × 10^6^	Transmembrane serine protease 4 (*TMPRSS*4)	Upstream of gene
	18	WU_10.2_18_60174495	54904058	8.71 × 10^6^	POU6F2	Intragenic
	8	MARC0056851	40158429	1.75 × 10^5^	factor interacting with PAPOLA and CPSF1 (*FIP1L1*)	Intragenic
	9	H3GA0027259	22298425	5.74 × 10^5^	glutamate metabotropic receptor 5 (*GRM5*)	Intragenic
	5	WU_10.2_5_67729569	65495186	9.52 × 10^5^	potassium voltage-gated channel subfamily A member 5 (*KCNA5*)	Upstream of gene
	12	ALGA0066694	23815963	9.62 × 10^5^	aminopeptidase puromycin sensitive (*NPEPPS*)	Intragenic
LA	10	WU_10.2_10_33859597	29938662	1.94 × 10^5^	neurotrophic receptor tyrosine kinase 2 (*NTRK2*)	Downstream of gene
	10	MARC0047936	34574312	6.16 × 10^5^	―	―
ALA	6	ASGA0091446	76942191	3.91 × 10^9^	*IGSF*21	Intragenic
	15	WU_10.2_15_134517097	121575947	1.23 × 10^5^	*OBSL*1	Intragenic
	8	MARC0056851	40158429	1.75 × 10^5^	*FIP1L1*	Intragenic
	6	WU_10.2_6_144009285	156191045	2.27 × 10^5^	phospholipid phosphatase 3 (*PLPP3*)	Upstream of gene
LA/ALA	18	WU_10.2_18_1597750	1630314	1.30 × 10^7^	Ubiquitin protein ligase E3C (*UBE*3*C*)	Intragenic
	18	WU_10.2_18_1255378	1239414	2.79 × 10^6^	Protein tyrosine phosphatase receptor type N2 (*PTPRN*2)	Intragenic
	11	ASGA0051239	62961302	4.68 × 10^6^	glypican 6 (*GPC6*)	Intragenic
	8	WU_10.2_8_2041192	2312933	9.48 × 10^6^	adrenoceptor alpha 2C (*ADRA2C*)	Downstream of gene
	6	ASGA0091446	76942191	3.59 × 10^5^	*IGSF*21	Intragenic

* indicates annotated gene at or closest to the SNP.

## Data Availability

The original contributions presented in the study are included in the article/Supplementary Materials, further inquiries can be directed to the corresponding author.

## Supplementary Materials

The following supporting information can be downloaded at https://www.mdpi.com/article/10.3390/foods13233927/s1: Table S1: The phenotypic statistics of backfat color and firmness in Beijing Black pigs; Table S2: The phenotypic value statistics of 32 fatty acids in Beijing Black pigs; Table S3: Annotated genes within 1 Mb upstream and downstream of significantly associated SNPs for DFAs; Table S4: Significant enrichment pathways; Table S5: Lipogenesis- and metabolism-related pathways.
