# Supplementary material for: Effect of Fatty Acids on Backfat Quality in Beijing Black Pigs
Source: Foods. 2024 Dec 5;13(23):3927. doi: 10.3390/foods13233927 (PMC11640767; doi:10.3390/foods13233927)
Supplement: Supplementary file 1 [file foods-13-03927-s001.zip › foods-3293145-supplementary.pdf]

Table S1 The phenotypic statistics of backfat colour and firmness in Beijing black pigs

Phenotypic	Number	Mean±sd	Max	Min
24h/b	488	5.40±0.75	7.74	3.45
24h/a	488	5.12±1.22	8.79	1.33
24h/L	488	85.55±1.61	90.09	79.71
Backfat firmness/N	339	198.12±54.40	359.15	64.13

Table S2. The phenotypic value statistics of 32 fatty acids in Beijing Black pigs

National standard name	**Common name**	**HG Mean±SD**	**LG Mean±SD**	**P-value**	**FC HG/LG**	**VIP**
Methyl butyrate	Butyric acid	0.02±0.00	0.02±0.00	0.35	0.95	0.29
Methyl caproate	Caproic acid	0.01±0.01	0.03±0.00	2.11×10^{7}	0.45	1.20
Methyl caprylate	Caprylic acid	0.03±0.00	0.04±0.00	1.20×10^{10}	0.65	1.29
Methyl hexanoate	Mallow acid	0.07±0.01	0.06±0.01	0.28	1.04	0.29
Methyl dodecanocarbonate	Lauric acid	0.07±0.01	0.07±0.01	0.12	1.06	0.42
Methyl tridecanoate	--------	0.02±0.00	0.02±0.00	4.77×10^{9}	0.69	1.24
Methyl tetradecanocarbonate	Myristic acid	0.86±0.12	0.55±0.05	9.79×10^{10}	1.55	1.26
Methyl cis-9-tetradecatetraenoate	Myristoleic acid	0.02±0.00	0.03±0.00	7.50×10^{5}	0.80	0.98
Methyl Pentadecanoate	--------	0.04±0.01	0.04±0	0.51	0.97	0.18
Methyl hexadecanoate	Palmitic acid	14.64±1.64	8.24±0.5	1.72×10^{14}	1.78	1.39
Methyl cis-9-hexadecenoate	Palmitoleic acid	1.11±0.29	0.77±0.1	0.00	1.44	0.91
Methyl seventeen carbonate	Pearsonic acid	0.16±0.06	0.12±0.02	0.02	1.35	0.62
Methyl cis-10-heptadecanoenoate	--------	0.13±0.04	0.1±0.02	0.01	1.36	0.69
Methyl octadecanocarbonate	Stearic acid	7.73±1.16	3.85±0.5	1.97×10^{12}	2.00	1.33
Methyl cis-9-octadecanoenoate	Oleic acid	10.83±1.14	6.29±0.73	2.53×10^{13}	1.72	1.37

trans,trans-9,12-Octadecadienoic acid methyl ester	trans-Linoleic acid	0.06±0.01	0.05±0.00	0.03	1.16	0.59
cis,cis-9,12-Octadecadienoic acid methyl ester	Linoleic acid	9.43±1.58	4.65±1.09	$2.02×10^{10}$	2.03	1.31
cis,cis,cis-6,9,12-Octadecatrienoic acid methyl ester	Gamma-linolenic acid	0.03±0.00	0.03±0.00	0.83	0.99	0.08
cis,cis,cis-9,12,15-Octadecatrienoic acid methyl ester	Alpha-Linolenic Acid	0.55±0.11	0.23±0.06	$2.36×10^{10}$	2.39	1.33
Methyl eicosanoate	Arachidonic acid	0.18±0.03	0.12±0.01	$3.50×10^{8}$	1.54	1.24
Methyl cis-11-eicosanoenoate		0.49±0.10	0.26±0.03	$5.25×10^{9}$	1.90	1.27
cis-11,14-Eicosadienoic acid methyl ester	Epigallic acid	0.45±0.07	0.23±0.04	$1.97×10^{11}$	1.99	1.38
cis,cis,cis-8,11,14-Eicosatrienoic acid methyl ester	Arachidonic acid	0.07±0.01	0.05±0.01	$1.28×10^{5}$	1.37	1.07
cis-5,8,11,14-Eicosatetraenoic acid methyl ester	ARA (arachidonic acid)	0.14±0.02	0.09±0.02	$2.01×10^{7}$	1.51	1.19
Methyl cis-11,14,17-eicosatrienoate	--------	0.1±0.01	0.05±0.01	$4.06×10^{12}$	1.91	1.39
Methyl cis-5,8,11,14,17-eicosapentaenoate	EPA	0.02±0.00	0.03±0.00	$8.75×10^{5}$	0.80	0.96
Methyl docosanoate	Behenic acid	0.04±0.01	0.05±0.01	0.00	0.84	0.84
Methyl cis-13-docosaenoate	Erucic acid	0.03±0.00	0.03±0.00	0.15	0.94	0.39
Methyl cis-13,16-docosadienoate	--------	0.03±0.00	0.03±0.00	0.20	0.95	0.33
Methyl tricosenate	--------	0.02±0.00	0.03±0.00	0.00	0.83	0.85
Methyl	--------	0.04±0.01	0.05±0.01	0.00	0.80	0.94

tetracosanoate						
Methyl cis-15-tetracosmonoate	Neurotic acid	0.04±0.01	0.03±0.00	0.04	1.25	0.54

Fatty acid content in units of fatty acid content per 100g of backfat, the same below.

Table S3 Annotated genes within 1Mb upstream and downstream of significantly associated SNPs for DFAs

DFAs	Genes name
CA	*MGLL、CYP27A1、ABTB1、TYR、NOX4、CDK12、MED1、FOLH1B、FBXL20、STAC2, CACNB1, PLXDC1, TRIM77, NAALAD2, FBXO47, CHORDC1, LASP1NB, LASP1, RPL23, C17orf98, CWC25, PIP4K2B, PSMB3, PCGF2, CISD3, MLLT6, EPOP, SRCIN1, ARHGAP23, SOCS7, GPR179, MRPL45, NPEPPS, KPNB1, TBX21, MPLKIP, CDK13, RALA, YAE1, POU6F2, VPS41, ZNF212, ZNF783, ZNF777, KRBA1, ZNF467, ZNF862, ATP6V0E2, LRRC61, RARRES2, SEC61A1, KBTBD12, PODXL2, MCM2, TPRA1, PLXNA1, CHCHD6, TXNRD3, CHST13, ACP3, DNAJC13, ACKR4, ACAD11, UBA5, NPHP3, TMEM108, EFCAB11, TDP1, KCNK13, PSMC1, NRDE2, CALM1, TTC7B, RPS6KA5, GPR68, CCDC88C, PPP4R3A, CATSPERB, TC2N, FBLN5, TRIP11, ATXN3, NDUFB1, CPSF2, CNOT9, PLCD4, BCS1L, RNF25, STK36, TTLL4, PRKAG3, WNT6, WNT10A, CDK5R2, FEV, CRYBA2, CFAP65, IHH, NHEJ1, SLC23A3, CNPPD1, RETREG2, ZFAND2B, ABCB6, ATG9A, ANKZF1, GLB1L, STK16, TUBA4A, DNAJB2, PTPRN, DNPEP, DES, SPEGNB, GMPPA, ASIC4, CHPF, TMEM198, OBSL1, INHA, STK11IP, SLC4A3*
SA	*RAB38,CTSC, GRM5,OSBPL7, MRPL10, LRRC46, SCRN2, SP6, SP2, PNPO, PRR15L, CDK5RAP3, NFE2L1, CBX1, SNX11, SKAP1, HOXB1, HOXB2, HOXB3, SPATA18, USP46, RASL11B, SCFD2, LNX1, CHIC2, GSX2, PDGFRA , SIK3, PAFAH1B2, SIDT2, TAGLN, PCSK7, RNF214, BACE1, CEP164, DSCAML1, TMPRSS13, IL10RA, SMIM35, TMPRSS4, SCN2B, JAML, MPZL3, MPZL2, CD3E, CD3D, UBE4A, ATP5MG, KMT2A, TMEM25, IFT46, ARCN1, PHLDB1, TREH, DDX6, CXCR5, BCL9L, UPK2, FOXR1, CENATAC, RPS25, TRAPPC4, SLC37A4, HYOU1, VPS11, HMBS, H2AX, DPAGT1, C2CD2L, HINFP, ABCG4, NLRX1, NHERF4, CCDC153, CBL, MPLKIP, CDK13, RALA, YAE1, POU6F2, VPS41, ZNF212, ZNF783, ZNF777, KRBA1, ZNF467, ZNF862, ATP6V0E2, LRRC61, RARRES2, VWF, NTF3, KCNA5, KCNA1, KCNA6, GALNT8, NDUFA9, AKAP3, DYRK4, RAD51AP1, C12orf4, FGF6, FGF23, TIGAR, CCND2, PARP11, PADI6, RCC2, ARHGEF10L, ACTL8, IGSF21, KLHDC7A, PAX7, TAS1R2, ALDH4A1,, IFFO2, UBR4, EMC1, MRTO4, AKR7A2, SLC66A1, CAPZB*

LA	*GOLM*1、*NAA*35、*AGTPBP*1、*NTRK*2、*SLC*28*A*3、*TMEM*215、*NDUFB*6、*TOPORS*、*RIGI*、*ACO*1
ALA	*SPATA18, USP46, RASL11B, SCFD2, LNX1, CHIC2, GSX2, PDGFRA, PADI6, RCC2, ARHGEF10L, ACTL8, IGSF21, KLHDC7A, PAX7, TAS1R2, ALDH4A1, IFFO2, UBR4, EMC1, MRTO4, AKR7A2, SLC66A1, CAPZB, CNOT9, PLCD4, BCS1L, RNF25, STK36, TTLL4, CYP27A1, PRKAG3, WNT6, WNT10A, CDK5R2, FEV, CRYBA2, CFAP65, IHH, NHEJ1, SLC23A3, CNPPD1, RETREG2, ZFAND2B, ABCB6, ATG9A, ANKZF1, GLB1L, STK16, TUBA4A, DNAJB2, PTPRN, DNPEP, DES, SPEGNB, GMPPA, ASIC4, CHPF, TMEM198, OBSL1, INHA, STK11IP, SLC4A3, C8B, C8A, FYB2, PRKAA2, PLPP3*
LA / ALA	*DOCK*9、*UBAC*2、*GPR*18、*GPR*183、*TM9SF*2、*CLYBL*、*ZIC*5、*ZIC*2、*PCCA*、*TMTC*4、*NALCN*、*VIPR*2、*DYNC2I*1、ESYT2、NCAPG2、PTPRN2、DNAJB6、UBE3C、MNX1、NOM1、LMBR1、SHH、PADI4、PADI6、RCC2、ARHGEF10L、ACTL8、IGSF21、KLHDC7A、PAX7、TAS1R2、ALDH4A1、IFFO2、UBR4、EMC1、MRTO4、AKR7A2、SLC66A1、CAPZB、CFAP99、RNF4、FAM193A、TNIP2、SH3BP2、ADD1、MFSD10、NOP14、GRK4、HTT、MSANTD1、RGS12、HGFAC、LRPAP1、ADRA2C、HMX1、CPZ、TRMT44、ACOX3、HTRA3、SH3TC1、ABLIM2、AFAP1、SORCS2

Table S4 Significant enrichment pathways

Pathways name	*P* value	Gene name
Alzheimer disease	0.019	*PSMB*3、*WNT*6、*GRM*5、*WNT*10*A*、*CALM1*、*TUBA*4*A*、*BACE*1、*NOX*4、*NDUFB*6、*NDUFB*1、*NDUFA*9
Hedgehog signaling pathway	0.026	*CCND*2、*SHH*、*IHH*
Glycosaminoglycan biosynthesis	0.026	*CHPF*、*CHST*13
Hypertrophic cardiomyopathy	0.035	*PRKAA*2、*DES*、*CACNB*1、*PRKAG*3
Pathways in cancer	0.039	*FGF*23、*RALA*、*CCND*2、*WNT*6、*FGF*6、*WNT*10*A*、*CALM*1、*RPS*6*KA*5、*TXNRD*3、*SHH*、*CBL*、*PDGFRA*
Melanogenesis	0.040	*WNT*6、*WNT*10*A*、*TYR*、*CALM1*
Glucagon signaling pathway	0.040	*PRKAA*2、*CALM*1、*PRKAG*3、*PPP*4*R*3*A*
Basal cell carcinoma	0.040	*WNT*6、*WNT*10*A*、*SHH*

Retrograde endocannabinoid signaling	0.049	*GRM*5、*MGLL*、*NDUFB*6、*NDUFB*1、*NDUFA*9

Table S5 Lipogenesis and metabolism related pathways

Pathways name	Gene name
energy metabolism	*ATP*6*V0E*2、*ATP*5*MG*、*NDUFB*6、*NDUFB*1、*NDUFA*9
amino acid metabolism	*ATP*6*V0E*2、*ATP*5*MG*、*FOLH*1*B*、*TYR*、*ALDH*4*A*1、*KMT*2*A*、*NDUFB*6、*PCCA*、*NDUFB*1、*NDUFA*9
glycan biosynthesis metabolism	*CHPF*、*DPAGT*1、*CHST*13、*GALNT*8
lipid metabolism	*MGLL*、*PAFAH*1*B*2、*CYP*27*A*1、*ACOX*3、*PLPP*3
immune system	*ATP*6*V0E*2、*H*2*AX*、*CD*3*E*、*TBX*21、*CD*3*D*、*C*8*A*、*C*8*B*
cardiovascular disease	*PRKAA*2、*CALM*1、*DES*、*CACNB1*、*PRKAG*3、*NDUFB*6、*NDUFB*1、*NDUFA*9
